# Molecular Simulation on Permeation Behavior of CH_4_/CO_2_/H_2_S Mixture Gas in PVDF at Service Conditions

**DOI:** 10.3390/polym14030545

**Published:** 2022-01-28

**Authors:** Houbu Li, Xuemin Zhang, Huifang Chu, Guoquan Qi, Han Ding, Xiong Gao, Jixing Meng

**Affiliations:** 1 State Key Laboratory of Performance and Structural Safety for Petroleum Tubular Goods and Equipment Materials, CNPC Tubular Goods Research Institute, Xi’an 710077, China; lihoubu@cnpc.com.cn (H.L.); qiguoquan@cnpc.com.cn (G.Q.); dinghan@cnpc.com.cn (H.D.); 2 School of Materials Science and Engineering, Chang’an University, Xi’an 710064, China; chuchu123456782021@163.com; 3 Shaanxi Yanchang Petroleum Northwest Rubber LLC, Xianyang 712023, China; gaoxiong.69@163.com; 4 Jiangsu Key Laboratory of Engineering Mechanics, Southeast University, Nanjing 210096, China; jxmeng@seu.edu.cn

**Keywords:** PVDF, permeation, sorption, diffusion, molecular simulation, oil and gas gathering and transportation

## Abstract

Reinforced thermoplastic composite pipes (RTPs) have been widely used for oil and gas gathering and transportation. Polyvinylidene fluoride (PVDF) has the greatest potential as a thermoplastic liner of RTPs due to its excellent thermal and mechanical properties. However, permeation of gases is inevitable in the thermoplastic liner, which may lead to blister failure of the liner and damage the safe operation of the RTPs. In order to clarify the permeation behavior and obtain the permeation mechanism of the mixture gas (CH_4_/CO_2_/H_2_S) in PVDF at the normal service conditions, molecular simulations were carried out by combining the Grand Canonical Monte Carlo (GCMC) method and the Molecular Dynamics (MD) method. The simulated results showed that the solubility coefficients of gases increased with the decrease in temperature and the increase in pressure. The adsorption isotherms of all gases were consistent with the Langmuir model. The order of the adsorption concentration for different gases was H_2_S > CO_2_> CH_4_. The isosteric heats of gases at all the actual service conditions were much less than 42 kJ/mol, which indicated that the adsorption for all the gases belonged to the physical adsorption. Both of the diffusion and permeation coefficients increased with the increase in temperature and pressure. The diffusion belonged to Einstein diffusion and the diffusion coefficients of each gas followed the order of CH_4_ > CO_2_ > H_2_S. During the permeation process, the adsorption of gas molecules in PVDF exhibited selective aggregation, and most of them were adsorbed in the low potential energy region of PVDF cell. The mixed-gas molecules vibrated within the hole of PVDF at relatively low temperature and pressure. As the temperature and pressure increase, the gas molecules jumped into the neighboring holes occasionally and then dwelled in the holes, moving around their equilibrium positions.

## 1. Introduction

**Figure 1:**
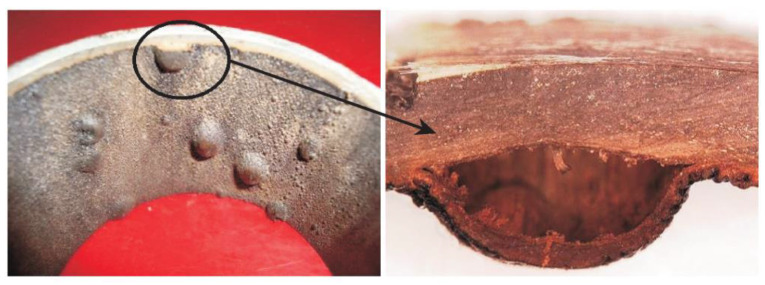
The blister failure of thermoplastic used in oilfield gathering and transportation.

Reinforced thermoplastic composite pipes (RTPs) with exceptional properties, such as corrosion resistance, flexibility, high strength, and fatigue resistance, are widely used for oil and gas transportation in the onshore and offshore petroleum industry [1]. They have a great advantage over conventional carbon steel pipelines. The principal components of RTPs includes three layers: thermoplastic as the inner lining layer, metal or non-metallic material as the reinforcing layer, and a thermoplastic protective layer applied on the outside [2]. High-density polyethylene (HDPE), polyamide (PA) and polyvinylidene fluoride (PVDF) are the most common thermoplastics used for the lining of RTPs to provide corrosion resistance against the transported medium. However, thermoplastics do not behave as perfect barriers to prevent gas permeation during gas transportation, especially in harsh conditions, such as high pressures and high temperatures. The gas in the transported medium is mainly constituted by methane but also contains carbon dioxide and hydrogen sulfide in varying amounts [3]. All these gases have a significant impact on the lifetime of the pipes [4,5]. Figure 1 shows a blister failure on the thermoplastic liner that has served China’s oilfields for several years. This blister failure will decrease the pressure-bearing capacity of the pipeline. It was revealed by the investigation that the failure might have resulted from gas permeation through the liner at high pressures and temperatures [6,7,8]. Therefore, understanding the permeation behavior and mechanism of the transported gases, such as CH_4_/CO_2_/H_2_S mixture in thermoplastics, has been a fundamental task in evaluating and controlling the gas permeability of RTPs in the oilfield.

Over the past years, experimental methods based on manometric measurements were applied to study gas permeability, diffusion, and solubility in the onshore and offshore petroleum industry. The gas permeation behavior of HDPE, which is the most used liner material of RTPs, was studied using the differential pressure method [9]. It was shown that the gas permeability coefficient of CO_2_ and CH_4_ at different temperatures through HDPE meets the Arrhenius equation. Flaconnèche et al. [10] applied the time lag method to obtain the transport coefficients of gases in PE, PA11 and PVF_2_. It has been found that the transport coefficients are independent of the membrane thickness. A continuous flow permeation method was used by Flaconnèche et al. [11] to determine the transport of CH_4_−CO_2_ gas mixtures through PE and polyvinylidene at pressures up to 100 bar. Some devices were developed to perform permeation tests at high pressures (1−100 MPa) and high temperatures (40 °C to 200 °C) on polymer samples (LDPE and MDPE) in the presence of gases such as CH_4_, CO_2_ and H_2_S [12]. Novel experimental apparatus and methodologies were also developed by Craster et al. [13] to quantify the transport of CO_2_ and chloride ions through polymer membranes under varying conditions of temperature and pressure and for prolonged periods of time. Pavani et al. [14] experimentally built and validated a device capable of providing reliable data on the transport coefficients of gases under extreme conditions. It has been found that the developed medium pressure gas permeameter can be utilized to qualitatively indicate the gas permeability of different materials, showing the difference in its permeability coefficient. However, measurements at higher pressure need extreme care and specific equipment and environment [15,16,17,18].

Compared with the experimental methods, computational simulation is an innovative approach which can predict material permeation properties and explore the mechanism at the molecular level. It also can quantify the influence of parameters, such as temperature and pressure, close to the gas transportation reality on the permeation coefficients. A lot of research has been devoted to calculating the solubility and diffusion of different gases in polymers by molecular simulation (MD). However, these works were mainly focused on the separation of gases by selective permeation or transport of fluids through polymer membranes, food packaging, and protective coatings [19,20,21,22]. Just a few researchers focused on gas permeation of thermoplastics in the oil–gas domain, especially that of the RTPs for hydrocarbon transport. However, gas permeation is an important issue for the oil and gas transportation system, especially under harsh conditions (as shown in Figure 1). Therefore, in order to propose an introduction on material selection and improvement of thermoplastic liners to prevent or reduce gas permeation, it is necessary to study the permeability characteristics and mechanisms of mixture gases in thermoplastics.

In this paper, the extruded PVDF pipe used as a liner of RTPs is a homopolymer with crystallinity of about 37%. Considering the crystalline phase as an impermeable phase, PVDF with an amorphous phase was taken as the matrix and CH_4_/CO_2_/H_2_S mixtures were considered as penetrants. The permeation behavior of gas molecules in PVDF was studied by molecular simulation systematically. The effects of temperature, pressure of actual gas transportation conditions on gas permeation behavior and mechanism were discussed, which were useful for the permeability evaluation and control of oil and gas in thermoplastics.

## 2. Theory

The permeation of gas molecules through polymers is typically demonstrated by solution-diffusion theory. Therefore, the permeability of gas molecules in polymers can be determined by two factors [23]. One is the gas solubility, which describes the gas concentration sorbed by polymers at the equilibrium, depending on the penetrant−polymer interactions. The other is gas diffusion, which describes the gas mass transport in polymers, reflecting the dynamics of the penetrant−polymer system. Therefore, the permeability coefficient *P* is given by the product of solubility coefficient *S* and diffusion coefficient *D*, as shown in Equation (1):

(1) P=S×D

The solubility coefficient *S* is related to the concentration *C* of gas in the polymer and the pressure *P_i_*. The relationship of *C* and *P_i_* can be expressed by a Dual Mode Sorption Model [24,25], as shown in Equation (2):

(2) $$C=K_{D}P_{i}+\frac{C_{H}P_{i}b}{1+P_{i}b}$$

where *K_D_* is the Henry constant, *C_H_* is the Langmuir adsorption capacity, *b* is the Langmuir constant. The unit of *C* is cm^3^ (STP)·cm^−3^.

By setting a certain temperature and pressure range, the curve of adsorption gas concentration *C* and pressure *P_i_* obtained by simulation, is the adsorption isotherm. When the pressure is zero, the limit slope is the solubility coefficient [26], as shown in Equation (3):

(3) $$S={\underset{P_{i}\to 0}{\lim}}_{}\frac{C}{P_{i}}=K_{D}+C_{H}b$$

The diffusion coefficient *D* is a measure of mean square molecular displacement. For sufficient lengths of time, the penetrant molecules perform random walks in the polymer matrix, the mean square displacement (MSD) becomes linear in time, and the diffusion coefficients can be calculated using the Einstein relation [27]:

(4) $$D=\frac{1}{6N}{\underset{t\to \infty }{\lim}}_{}\frac{d}{d_{t}}\left(\overset{N}{\underset{i=1}{\sum }}{\left[r_{i}\left(t\right)-r_{i}\left(0\right)\right]}^{2}\right)$$

where *D* is the diffusion coefficient, *N* is the total number of penetrants in the system, *r*(*t*) is the penetrant’s position at time t, *r*(0) is its initial position, and ${\left[r_{i}\left(t\right)-r_{i}\left(0\right)\right]}^{2}$ is the MSD of the molecule, denoting the ensemble average. Since the MSD value has averaged the position changes of *N* penetrants in the system, *a* is taken as the slope of the MSD curve obtained by molecular simulation. Therefore, Equation (4) can be simplified as Equation (5):

(5) D=a/6

Independent determinations of *S* and *D* are possible from molecular simulations. In this study, the Grand Canonical ensemble Monte Carlo (GCMC) and Molecular Dynamics (MD) simulation were applied to estimate the solution coefficients and diffusion coefficients of penetrant CH_4_/CO_2_/H_2_S mixture gases over a wide range of temperatures and pressures. GCMC and MD simulations also offered insight into the molecular mechanisms of penetrant gases transported through the thermoplastics.

## 3. Model and Simulation Methodology

### 3.1. Establishment of the Molecule Models

The structures of gas molecules and the PVDF chain were generated by the visualizer module of Materials Studio (MS). The gas molecular models, such as CH_4_, CO_2_, and H_2_S, were built by a draw tool separately. The PVDF chain was constructed by a homopolymer tool with vinylidene fluoride as repeat unit with the chain length of 10. Then, the PVDF chain and gas molecules were geometry optimized using the smart minimizer in the Forcite module.

### 3.2. Simulations of Gas Sorption in PVDF

The solubility of the CH_4_/CO_2_/H_2_S gas mixture in PVDF was simulated by GCMC simulation method, which has been widely used for many studies of adsorption processes [28,29,30]. The GCMC simulation method aims to describe the polymer in equilibrium with gases at a given temperature (T) and pressure (P) [3]. In this study, temperatures and pressures were selected for GCMC simulation according to the actual service conditions of RTPs in an oilfield; that is, the temperatures were set at 30 °C, 40 °C, 60 °C, and 80 °C, and the pressures were set at 2.5 MPa, 6.4 MPa, 8.5 MPa, and 10 MPa.

**Figure 2:**
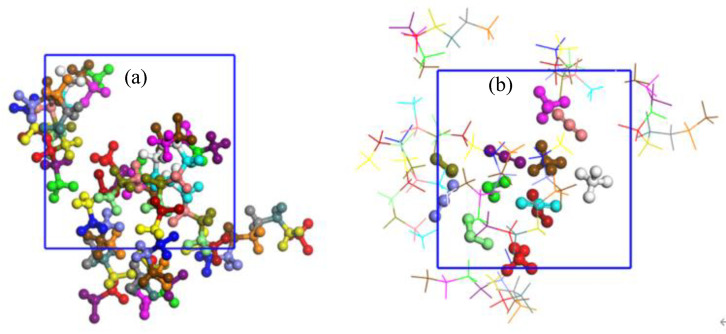
The stable configuration of sorption cell model (**a**) and diffusion cell model (**b**).

The initial sorption cell of PVDF with four chains after geometry optimization was constructed with the Amorphous Cell module. The cell was first exposed to the energy minimization to eliminate local non−equilibrium. Thereafter, the 200 ps canonical ensemble (NVT) and 200 ps isothermal−isobaric ensemble (NPT) MD simulations were performed successively to relax the polymer structure and obtain the final equilibrated structure. The resulting relaxed configuration was then used as a starting structure for sorption simulations, as shown in Figure 2a.

**Figure 3:**
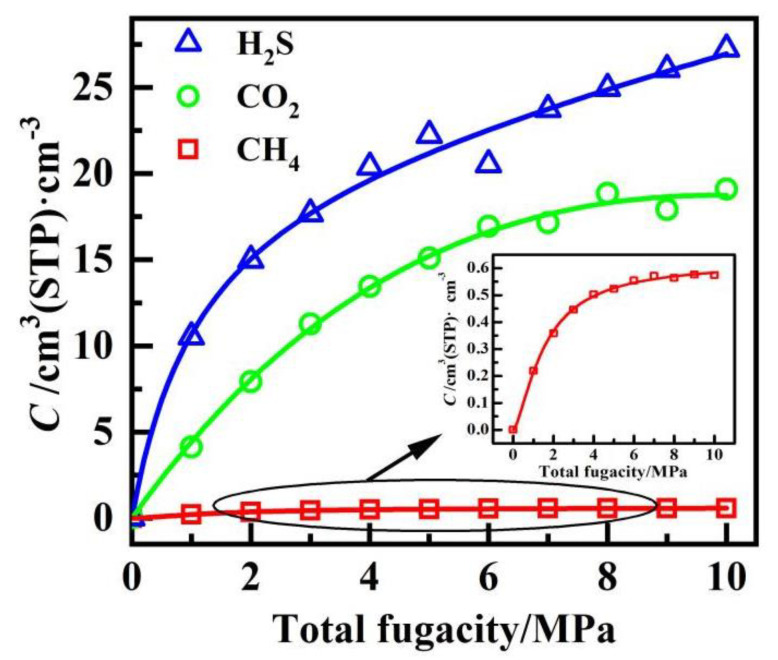
Adsorption isotherms of CH_4_/CO_2_/H_2_S mixed gases in PVDF at 30 °C.

The PVDF unit cell with stable structure (Figure 2a) was used as an adsorbent while the optimized mixture gas molecules were used as adsorbates in the Sorption module of Materials Studio (MS). By using an isothermal adsorption task at a certain temperature and 10 isotherm points of fugacity in the pressure from 0 to 10 MPa, the adsorption isotherm can be obtained (Figure 3) and then the solubility coefficients can be calculated. The fixed pressure task was used to obtain isosteric heat. The density field distribution and isopycnic of each gas were obtained by the Volumetric Selection module.

During the simulation, the COMPASS force field was employed, and the Metropolis algorithm was adopted. The electrostatic interactions were handled by Ewald electrostatic and group−based for van der Waals interactions. The thermostat parameter was Nose and the Barostat was Berendsen. At each temperature and pressure, 1 × 10^6^ steps of calculations were performed using the equilibration steps period of 1 × 10^5^ steps.

### 3.3. Simulations of Gas Diffusion in PVDF

The Molecular Dynamics (MD) method [31,32,33,34] was used to simulate the diffusion process of gases in PVDF. The diffusion cell of PVDF and CH_4_/CO_2_/H_2_S gas mixture molecules was constructed with the Amorphous Cell module. Four PVDF chains and four molecules of each gas were placed into the cell, in which the position and the orientation of the molecules were randomly assigned. The Smart Minimizer was used to minimize the energy and a 500 ps NPT simulation was performed to obtain a stable configuration, as shown in Figure 2b. Then, an NVT simulation was carried out at certain conditions to obtain the motion behavior of the molecules.

The Analysis task of Materials Studio 7.0 (Accelrys Software Inc., San Dieg, CA, USA) was used to obtain the atomic trajectories and MSD curve. The initial rate of each molecule was randomly generated by Maxwell−Boltzmann distribution. The Nose method was used to control the temperature and the Berendsen method was used to maintain the pressure at a certain target. All of the temperature and pressure conditions were set as shown in Section 3.2. During non−bonding interactions, the group−based method was used for the van der Waals interaction, and the Ewald method was used for electrostatic interactions. The simulation time was 500 ps with a time step of 1.0 fs. Output information of the system was recorded every 1000 steps.

## 4. Results and Discussions

### 4.1. Sorption of Gas Mixture in PVDF

#### 4.1.1. Solubility Coefficients and Isosteric Heats of CH_4_/CO_2_/H_2_S Mixture Gases

**Figure 4:**
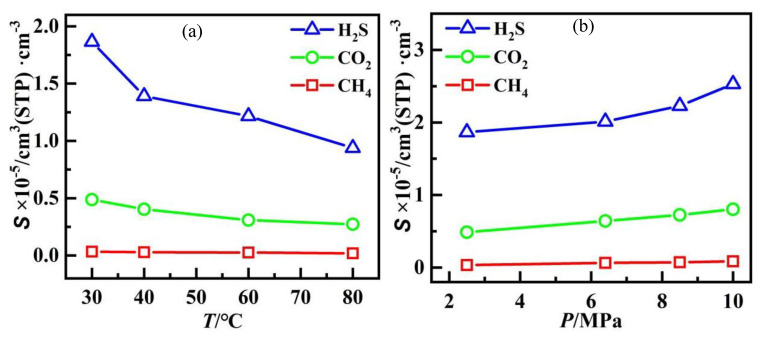
The solubility coefficients of gases at different conditions: (**a**) 2.5 MPa; (**b**) 30 °C.

The adsorption isotherm of CH_4_/CO_2_/H_2_S mixture gases at 30 °C under the pressure of 0−10 MPa is shown in Figure 3. It can be seen that the adsorption gas concentration *C* of all gases increases constantly with pressure, and the fit of the *C*−*P* curves of H_2_S, CO_2_ and CH_4_ with the Langmuir model were 0.989, 0.995, and 0.998, respectively, corresponding to the Langmuir single−layer reversible adsorption. However, the concentration of CH_4_ molecules adsorption was very low and remained almost unchanged with increasing pressure. According to Equation (3), the solubility coefficients of each gas under different conditions were obtained by fitting the adsorption isotherms with the nonlinear least square method, as shown in Figure 4. The same trend can be observed whatever the gas, that is, the solubility of gases, decreases with increasing temperature and decreasing pressure. However, the changing of solubility for CH_4_ is not obvious. Furthermore, the concentration of H_2_S adsorption was much larger than that of CO_2_ and CH_4_ under the same temperature and pressure, which indicates a significantly competitive advantage. The solubility followed the order of H_2_S > CO_2_ > CH_4_ for all the test conditions.

**Figure 5:**
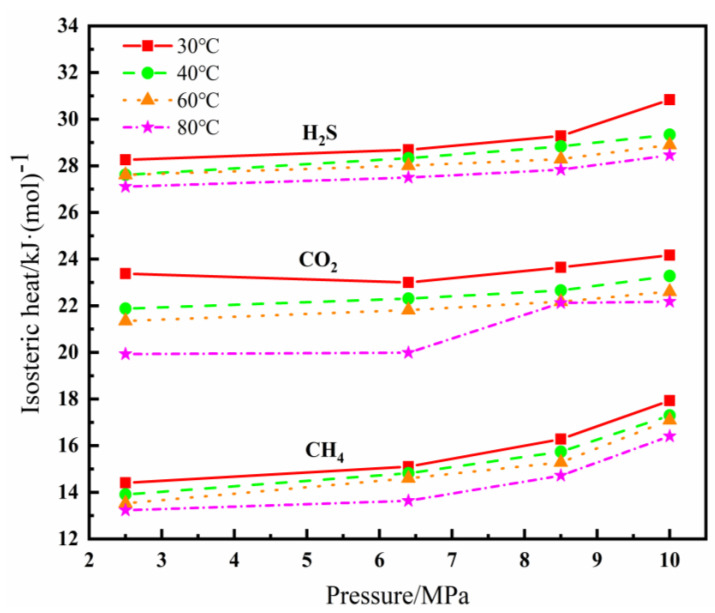
Isosteric heat of adsorption of gas molecules in PVDF at different conditions.

The isosteric heat (Q_st_) is one of the important indexes to measure the adsorption function of the adsorbent, which reflects the intensity of the adsorption progress. Generally, the higher the isosteric heat, the stronger the adsorption is [35]. Figure 5 shows the isosteric heat of adsorption of CH_4_, CO_2,_ and H_2_S at different conditions. The isostericheat increases as the pressure increases and the temperature decreases. It indicates that the interactions of PVDF molecules and adsorbates were gradually strengthened as above conditions change, which is consistent with the variation trend of the solubility coefficients. All the heat values were less than 42 kJ/mol, suggests that the adsorption of PVDF to gases belongs to the physical adsorption [36,37]. Moreover, the isosteric heat of three gases at all test conditions showed a pattern of H_2_S > CO_2_ > CH_4_.

#### 4.1.2. Density Field Distributionand Isopycnic

**Figure 6:**
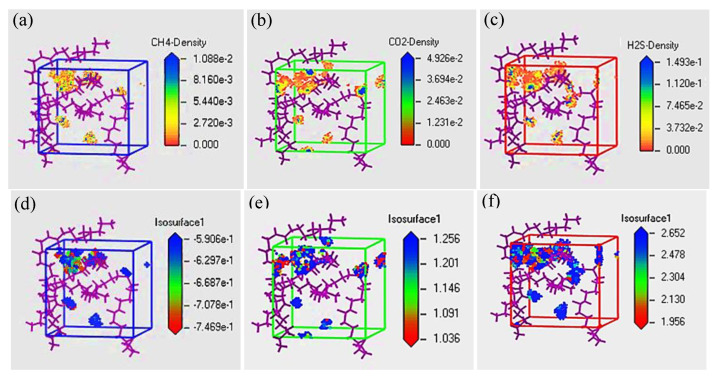
Density field distribution and isopycnic of CH_4_ molecule (**a**,**d**); CO_2_ molecule (**b**,**e**); H_2_S molecule (**c**,**f**) in simulated cell at 2.5 MPa and 30 °C.

The density field distributions and isopycnic of gas molecules dissolved in PVDF at 30 °C and 2.5 MPa are shown in Figure 6. The scatter regions represent the positions where the dissolved gas molecules are distributed in the PVDF cell (Figure 6a−c), exhibiting that the adsorption of gas molecules in PVDF is selective aggregated rather than uniform adsorption. The isopycnics responding to the density field distributions are shown in Figure 6d−f. The colors on the isopycnic describe the corresponding energy field value. The blue zones (with large numerical value) represent the high intermolecular interaction energy in the cell, while the red zones(with small numerical value) represent the low one. Therefore, during the simulation process, the red zone is the “hot spot” of gas molecular adsorption (i.e., the most easily adsorbed position), which is consistent with the density field distribution. In addition, on the basis of the scatter regions, density and energy field values shown in Figure 6, it can be concluded that the gas adsorption capacity followed H_2_S > CO_2_ > CH_4_, and the aggregation of H_2_S molecules was more compact than those of CO_2_and CH_4_.

### 4.2. Diffusion of Gas Mixture in PVDF

#### 4.2.1. Diffusion Coefficients and Fractional Free Volume of CH_4_/CO_2_/H_2_S Mixture Gases

**Figure 7:**
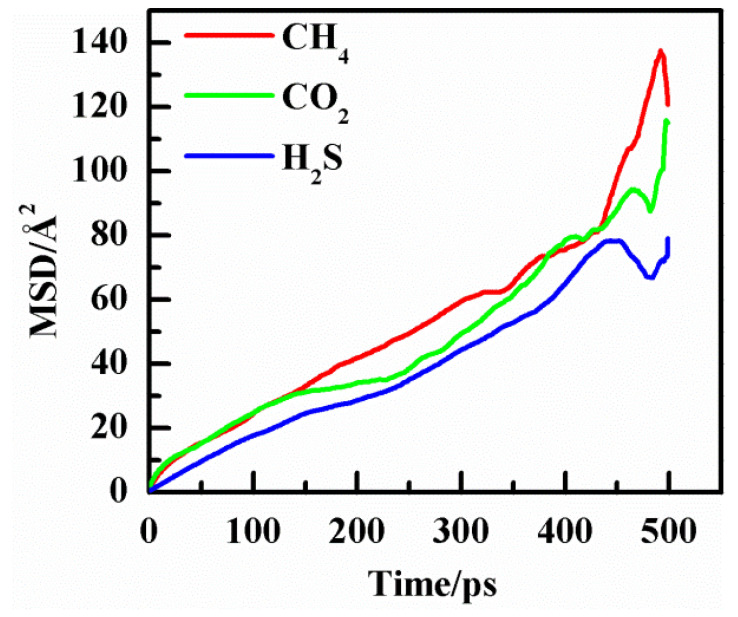
Mean square displacement curve of gases in PVDF at 2.5 MPa and 30 °C.

**Figure 8:**
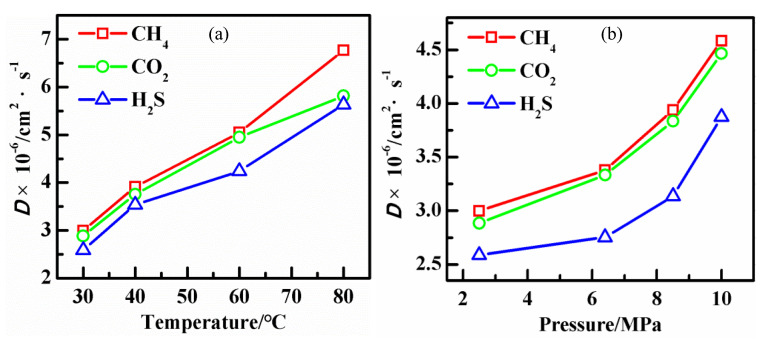
The diffusion coefficients of gases at different conditions: (**a**) 2.5 MPa; (**b**) 30 °C.

Mean square displacement (MSD) analysis was a technique to determine the mode of displacement of particles followed over time [32]. Examination of the MSD can provide valuable information about the diffusion process of molecules. Figure 7 shows MSD curves of CH_4_/CO_2_/H_2_S molecules dissolved in PVDF at 2.5 MPa and 30 °C. The value of MSD increases with the time *t* increasing, showing a linear relationship. At the end of the curves, noisy points appear due to statistic error and need to be eliminated. The diffusion coefficient of gas molecules can be calculated with the linear part of the MSD curves. Therefore, the diffusion coefficients were calculated from Equation (5) and then calculated diffusion coefficients of gases at different conditions are shown in Figure 8. It can be seen that the diffusion coefficients of the three gases increase by increasing temperature and pressure, which is in agreement with the results of the former work [3]. It also reveals that the diffusion coefficients for all three gases are as follows: CH_4_ > CO_2_ > H_2_S.

According to Fox and Flory’s free volume theory, the volume of a polymer consists of the volume occupied by polymer and the free volume that is not occupied by polymer in the form of “holes”. The free volume provides sufficient space for the movement of polymer chains and the diffusion of small molecules, whose size is directly related to the diffusion coefficient [38,39]. The free volume of the PVDF−gas mixture system was calculated by the Atom Volume and Surface module in MS, as shown in Table 1. It is noted that the fractional free volume increases with the increase in temperature and pressure, which means that the hole free volume will be redistributed faster and penetrated molecules have higher energy to overcome the activation energy required to jump into new holes [23]. More free volume can increase the probability of gas molecule jumps, thereby improving the diffusion ability of gas molecules. Therefore, the diffusion coefficients increase with the increasing of temperature and pressure.

#### 4.2.2. Diffusion Trajectory and Displacement

**Figure 9:**
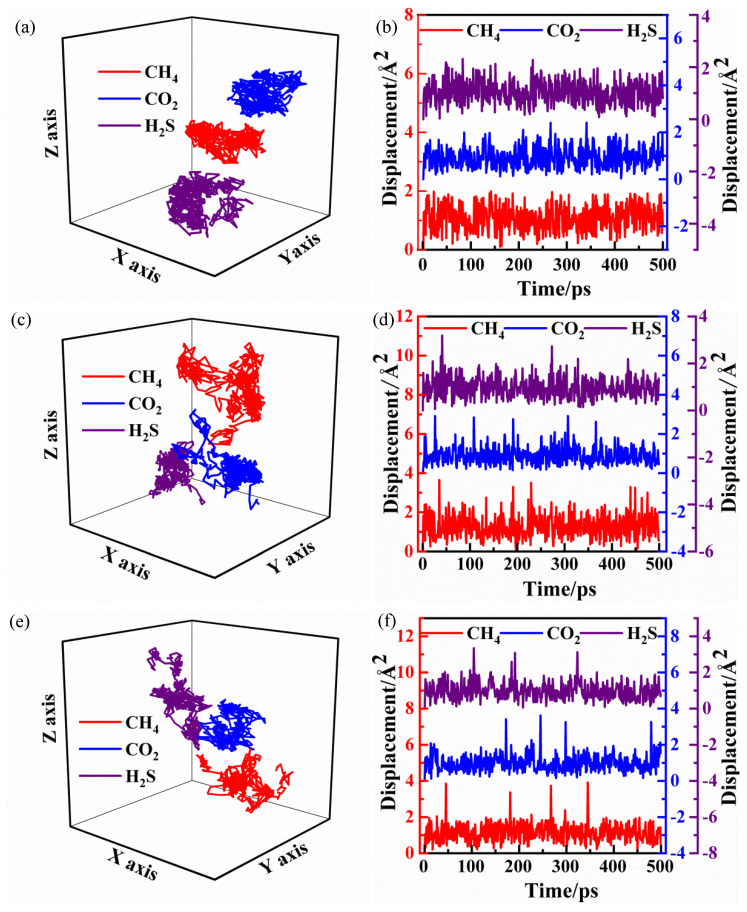
The diffusion trajectory and displacement of single gas molecules in PVDF under different conditions in CH_4_/CO_2_/H_2_S mixed gas medium: (**a**,**b**) 2.5 MPa, 30 °C; (**c**,**d**) 2.5 MPa, 40 °C; (**e**,**f**) 6.4 MPa, 30 °C.

The three-dimensional diffusion trajectory and displacement of individual gas molecules in PVDF under different conditions are shown in Figure 9. It can be seen that gas molecules of CH_4_, CO_2_ and H_2_S vibrate in existing holes for a considerable time under the condition of 2.5 MPa and 30 °C (Figure 9a), and the displacement of motion is always limited within 0.1 nm (Figure 9b). When the pressure is constant and the temperature is increased from 30 °C to 40 °C, the diffusion trajectories of each gas molecule become more scattered (Figure 9c), and the molecules jump into the neighboring holes occasionally. The jumping frequencies of 6, 5 and 4 are obtained in the range of 0−500 ps for CH_4_, CO_2_ and H_2_S, respectively. The jumping distance is around 0.2 nm, and the diffusion ability becomes stronger (Figure 9d). As the pressure is increased to 6.4 MPa, the diffusion trajectories of gas molecules are no longer concentrated (Figure 9e), which indicates that the higher the pressure is, the faster the movement of molecules can be observed. Although there are 5, 4, and 4 large-scale jumps in CH_4_, CO_2_, and H_2_S molecules at this condition, the jumping distance is obviously increased, which is more than 0.25 nm (Figure 9f). After each long-distance transition, the gas molecules will jump to the adjacent holes and continue to vibrate around their equilibrium positions. Therefore, it can be concluded that the diffusion of gas molecules belongs to Einstein diffusion.

### 4.3. Permeability Coefficients and Mechanisms

**Figure 10:**
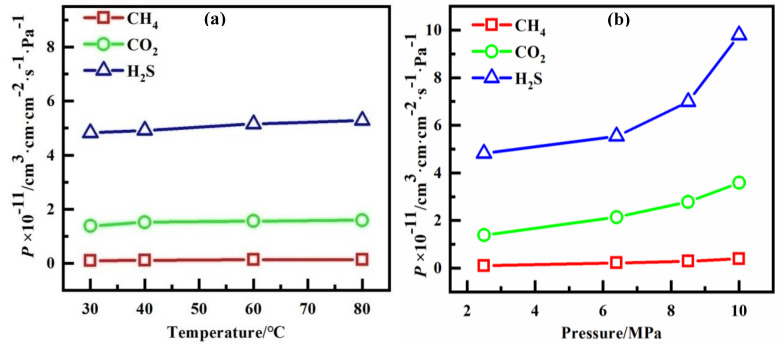
The permeability coefficients of gases at different conditions: (**a**) 2.5 MPa; (**b**) 30 °C.

The permeability can be calculated by Equation (1), based on the calculated solubility coefficients and diffusion coefficients in this work. As shown in Figure 10, the permeability coefficients of all gases increase lightly with increasing temperature (Figure 10a), which indicates that high temperature has a limited effect on permeation. At the same temperature, the permeability coefficients almost doubles as the pressure increases from 2.5 MPa to 10 MPa (Figure 10b). Furthermore, PVDF is much more permeable with H_2_S, compared to other gases in this work, because of the higher solubility coefficients of H_2_S in PVDF and its relatively higher diffusion coefficient.

Based on the above sorption and diffusion analysis, the permeation mechanism of gas molecules in PVDF can be concluded as follows: first, the adsorption of gas molecules in PVDF shows selective aggregation, and most of them are adsorbed in the low potential energy region of the unit cell. Then, the gas molecules adsorbed in PVDF makes movement within the hole in a small amplitude vibration at low temperature and low pressure. As the temperature and pressure increase, the gas molecules jump into the neighboring holes from time to time and then dwell in the holes, moving around their equilibrium positions.

According to the above results, it can be concluded that the molecular simulation method can quickly and effectively analyze the gas permeability in PVDF. Temperature has a limited influence on gas permeability, while pressure has a greater influence. Thus, PVDF lined RTPs can be safely used in gas containing conditions with higher temperature and lower pressure. Moreover, the permeability of H_2_S is far greater than that of CO_2_ and CH_4_ according to the above simulation results, a reminder that more attention should be attracted regarding RTPs used in H_2_S−containing conditions. Based on analysis of the diffusion mechanism, the free volume which exists in the amorphous region of the polymer is the main channel of gas diffusion. Therefore, adjusting the crystallinity of PVDF by improving its extrusion process can control the proportion of the amorphous region, and then reduce the possibility of gas permeability as well as blistering of the PVDF liner in RTPs.

## 5. Conclusions

In this work, the Grand Canonical Monte Carlo method and a molecular dynamics simulation were performed to study the permeation behavior and the molecular−level mechanism of CH_4_/CO_2_/H_2_S gas molecules in PVDF. The results showed that:

The solubility coefficients of each gas molecule in PVDF increased with the decrease in temperature and the increase in pressure and the order of solubility was H_2_S > CO_2_> CH_4_. The isosteric heats of gases for the normal service conditions were much less than 42 kJ/mol, which indicates that the adsorption for all the gases belong to physical adsorption.

The diffusion coefficients increased with the increase in temperature and pressure. The diffusion of gas molecules in PVDF belonged to Einstein diffusion and the diffusion capacity of each gas was in the order of CH_4_ > CO_2_ > H_2_S.

The permeability coefficients increased with the increase in temperature and pressure. The adsorption of gas molecules in PVDF was selective aggregation and the diffusion of the gas molecules in PVDF was Einstein diffusion. The diffusion process indicated that the molecules dwell in the existing holes in PVDF for a considerable time and occasionally jump into the neighboring holes. As the temperature and pressure increase, the gas molecules jumped into the neighboring holes from time to time and then dwelled in the holes, moving around their equilibrium positions.

## Figures and Tables

**Table 1:** Fractional free volume of gas molecules in PVDF at different conditions.

	Temperature/°C	30	40	60	80
Pressure/MPa					
2.5		0.303	0.323	0.330	0.342
6.4		0.321	0.332	0.342	0.355
8.5		0.331	0.345	0.351	0.369
10		0.344	0.350	0.357	0.383

## Data Availability

The data presented in this study are available on request from the corresponding author.
